# Epidemiology and risk factors of soil-transmitted nematode-schistosome co-occurrence: An analysis of the global burden of disease study

**DOI:** 10.1371/journal.pntd.0014224

**Published:** 2026-05-04

**Authors:** Ruoyao Wang, Bin Le, Chao Chen, Huiyu Xia, Xiaobin Fan, Chao Zhu, Xing He

**Affiliations:** 1 Department of Tropical Diseases, Naval Medical University, Shanghai, China; 2 Department of Laboratory Animal Science, Naval Medical University, Shanghai, China; 3 Department of Rheumatology and Immunology, Shanghai Eastern Hepatobiliary Surgery Hospital, Naval Medical University, Shanghai, China; Instituto de Salud Carlos III, SPAIN

## Abstract

**Objective:**

Soil-transmitted nematode (STN) infections and schistosomiasis frequently co-occur in endemic regions, imposing substantial combined health burdens. This study aimed to characterize the global co-occurrence patterns, long-term trajectories, and key risk factors of STN-schistosome co-occurrence using Global Burden of Disease (GBD) data.

**Methods:**

We analyzed GBD 1990–2021 data from 69 countries and territories with available STN infections and schistosomiasis prevalence data. Co-occurrence patterns were classified into consistent, schistosomiasis-dominant, and STN infection-dominant regions based on global prevalence quartiles. Group-based trajectory modeling (GBTM) and similarity network clustering identified long-term evolutionary trajectories and epidemiological clusters. Negative binomial regression and population-attributable fraction (PAF) analysis quantified associations between 22 risk factors and disease burden.

**Results:**

Global co-occurrence patterns remained stable over 32 years, with 68.1% of countries and territories showing concordant high or low burdens of both diseases. GBTM identified three distinct trajectories for each disease, with sub-Saharan Africa dominating high-burden groups. Similarity network clustering partitioned countries into six epidemiological clusters, ranging from persistently high co-burden to near-eliminated schistosomiasis with low STN prevalence. Key shared drivers included inadequate water, sanitation, and hygiene (WASH) factors, while nutritional deficiencies (iron deficiency, child growth failure) were specific risk factors for STN infections, particularly in co-endemic regions (PAF = 19.08% and 8.82%, respectively). Both high and low temperatures exerted protective effects against both infections.

**Conclusion:**

STN-schistosome co-occurrence exhibits distinct global epidemiological clusters with heterogeneous drivers. Integrated control strategies should combine WASH improvements, preventive chemotherapy, and targeted nutritional interventions, especially in co-endemic regions, to align with the WHO 2021–2030 Neglected Tropical Diseases Roadmap and accelerate elimination efforts.

## Author summary

Soil-transmitted nematodes (roundworm, whipworm, hookworm) and schistosomes affect over 1.5 billion people worldwide, predominantly in impoverished communities across Africa, Asia, and Latin America. These parasites frequently occur together in the same regions, yet we lack a comprehensive understanding of their global co-occurrence patterns and shared drivers. In this study, we analyzed 32 years of data from the Global Burden of Disease Study across 69 countries to characterize where and why these infections co-occur. We identified six distinct global patterns of co-occurrence, ranging from persistently high-burden regions in sub-Saharan Africa to areas that have nearly eliminated schistosomiasis. Our findings reveal that inadequate water, sanitation, and hygiene (WASH) are common drivers for both parasites, while nutritional deficiencies—particularly iron deficiency and child growth failure—disproportionately increase soil-transmitted nematode burden, especially in regions where both parasites coexist. These results suggest that integrated control strategies combining deworming programs with improved sanitation and nutritional support would be more effective than single-disease approaches. By identifying priority regions and modifiable risk factors, our work provides actionable evidence to guide global efforts toward the World Health Organization’s 2030 targets for neglected tropical disease elimination.

## Introduction

Soil-transmitted nematodes (STNs) and schistosomes are two groups of neglected tropical parasites that impose substantial global health burdens, particularly in low- and middle-income countries across Africa, Asia, and Latin America. Globally, an estimated 1.5 billion people are infected with at least one STN species (*Ascaris lumbricoides*, *Trichuris trichiura*, hookworms), while approximately 240 million require preventive chemotherapy for schistosomiasis [1–3]. A defining epidemiological feature of these parasites is their frequent co-occurrence in endemic settings—driven by shared transmission pathways linked to inadequate water, sanitation, and hygiene (WASH) conditions, agricultural practices, and socioeconomic vulnerabilities [4–7]. Field studies have consistently documented high co-infection rates in local communities, highlighting that polyparasitism is not an incidental finding but a prevalent public health challenge in resource-constrained regions [8–10].

Co-occurrence with STNs and schistosomes poses unique threats to human health that transcend the impacts of single infections. Evidence suggests that concurrent parasitic exposure can exacerbate clinical outcomes, including increased anemia severity, impaired childhood growth and cognitive development, and reduced responsiveness to treatment [11,12]. Beyond individual health impacts, the co-endemicity of these parasites complicates public health interventions. Mathematical modeling studies have emphasized that co-infection dynamics alter transmission patterns and treatment efficacy, underscoring the limitations of single-disease control strategies in polyparasitic settings [13]. Despite this, most epidemiological research has focused on individual parasite species, leaving critical gaps in our understanding of global co-occurrence patterns, their temporal dynamics, and region-specific risk profiles.

While localized studies have provided valuable insights into co-infection prevalence at the community level, they lack the geographic scope and temporal depth needed to inform global and regional policy. The Global Burden of Disease (GBD) Study offers a unique opportunity to address this limitation, as it provides standardized, population-level data on disease burden and risk factors across more than 200 countries and territories over decades [14,15]. Recent GBD-based research on comorbid non-communicable diseases has demonstrated the power of this database to uncover global co-occurrence patterns and identify high-priority regions for targeted intervention [16,17]. However, analogous analyses for STN-schistosome co-occurrence remain scarce, with existing global parasitic disease research often focusing on single pathogens or aggregate burden metrics rather than co-morbidity dynamics.

To fill this gap, the present study leverages GBD data (1990–2021) to systematically characterize the global co-occurrence patterns of STN and schistosome infections, their temporal trajectories, and key driving risk factors. Findings from this study will provide a comprehensive global portrait of STN-schistosome co-occurrence, informing the development of integrated, region-tailored control strategies aligned with the WHO Neglected Tropical Diseases Roadmap (2021–2030) and addressing the unmet need for data-driven polyparasitism management.

## Methods

### Overview

This study systematically explored the global co-occurrence patterns, long-term trajectory characteristics, and associated risk factors of schistosomiasis and STN infections using comprehensive data from the GBD Study 1990–2021. A combined analytical framework integrating cross-sectional and longitudinal approaches was adopted to ensure robust identification of stable co-occurrence modes and dynamic evolutionary trends. Advanced statistical and computational methods, including negative binomial regression, population-attributable fraction (PAF) estimation, group-based trajectory modeling (GBTM), and similarity network clustering, were applied to quantify associations between risk factors and disease burden, classify long-term prevalence trajectories, and identify country clusters with similar epidemiological characteristics. This study followed the GATHER guidelines [18].

### Study population and variables

Data were extracted from the GBD 2021 databases, covering 204 countries and territories worldwide. All the countries and territories were listed in S1 Table. The core outcome variables were the age-standardized prevalence rates of schistosomiasis and STN infections (including *Ascaris lumbricoides*, *Trichuris trichiura*, and hookworm) among the general population. Exposure data for 22 potential risk factors were retrieved, categorized into three functional groups: WASH factors (e.g., unsafe water access), environmental factors (e.g., high temperature), and nutritional factors (e.g., iron deficiency). All risk factor exposures were quantified using the summary exposure value (SEV) as defined in the GBD database, ensuring standardized comparison across countries and time periods.

### Construction of global distribution maps

The maps were generated using the maps R package (https://github.com/adeckmyn/maps; version 3.4.3). Base map data (country or region borders) used in this R package are based on public domain data from the Natural Earth project (https://www.naturalearthdata.com/; terms of use: http://www.naturalearthdata.com/about/terms-of-use/).

### Definition of co-occurrence patterns

To characterize the spatial synergy and divergence of schistosomiasis and STN infection burdens, the age-standardized prevalence rates of both diseases were independently categorized into four levels based on global quartiles: low (<25th percentile), lower-middle (25th–50th percentile), upper-middle (50th–75th percentile), and high (>75th percentile). Countries and territories were then classified into three distinct co-occurrence patterns by comparing the relative levels of the two diseases: the consistent pattern, where the prevalence levels of schistosomiasis and STN infections were identical; the schistosomiasis-dominant pattern, where the prevalence level of schistosomiasis was higher than that of STN infections; and the STN-dominant pattern, where the prevalence level of STN infections was higher than that of schistosomiasis. This classification served as the foundation for subsequent stratified analyses of risk factors and disease burden attribution.

### Key risk factor quantification

Negative binomial regression models were constructed at the country level to quantify the association between each risk factor’s SEV and the prevalence of schistosomiasis or STN infections [19]. Relative risk (RR) and 95% confidence intervals (CIs) were calculated to assess the strength of associations, with statistical significance set at *P* < 0.05.

### Population-attributable fraction (PAF) analysis

To evaluate the public health impact of identified key risk factors, PAF was computed to estimate the proportion of disease burden theoretically preventable by eliminating exposure to each factor: single-factor PAF, which is calculated using the formula: PAF=(RR − 1)/RR, with RR denoting the relative risk derived from negative binomial regression [20]; stratified PAF analysis, which entails separately computing PAF for the three co-occurrence pattern groups (consistent, schistosomiasis-dominant, STN infection-dominant) to explore variations in risk factor contributions across different epidemiological settings; and combined PAF (cPAF), which estimates the joint attribution of all key risk factors to each disease using the formula:$cPAF=1-\prod_{i=1}^{n}{(1-PAF}_{i})$, and reflects the maximum potential disease burden reduction achievable through comprehensive risk factor interventions. In this formula, $\prod_{i=1}^{n}\left(1-{PAF}_{i}\right)$ is the product of the complements of individual PAFs, used to calculate the probability of the disease burden not being attributable to these risk factors. ${PAF}_{i}$ is the population attributable fraction of the specific risk factor in the i-th risk country or territory, denoting the proportion of disease that can be reduced by eliminating this factor alone, and n represents the total number of places included in the analysis.

### Group-based trajectory modeling (GBTM) analysis

GBTM was applied to the 32-year (1990–2021) prevalence time-series data of each country to identify typical long-term evolutionary trajectories of schistosome and STN co-occurrence. Models with different polynomial orders (linear, quadratic, cubic) and trajectory group numbers (3–6 groups) were compared using information criteria (akaike information criterion, AIC; bayesian information criterion, BIC), with the optimal model selected based on the lowest AIC and BIC values [21]. Trajectories were categorized into distinct types (e.g., persistently high, rapidly declining, stable low, gradually increasing) to quantify and classify country-specific long-term epidemiological trends, providing a basis for predictive modeling and targeted intervention planning.

### Similarity network clustering analysis

A country-to-country similarity matrix was constructed based on two key indicators: the 32-year mean prevalence of both diseases and the trajectory characteristics identified by GBTM. The matrix was converted into an undirected weighted network, where edge weights represented the similarity between pairs of countries. The Louvain community detection algorithm was applied to this network to identify clusters of countries with similar disease burden evolution patterns [22]. This approach transcended geographical proximity to reveal hidden global epidemiological structures and explore associations between these clusters and geographical, socioeconomic, and environmental factors.

### Statistical software

All statistical analyses and data visualizations were performed using R version 4.5.1 (R Foundation for Statistical Computing, Vienna, Austria) with packages.

## Results

### Global co-occurrence patterns of the two parasitic diseases in 2021

**Fig 1:**
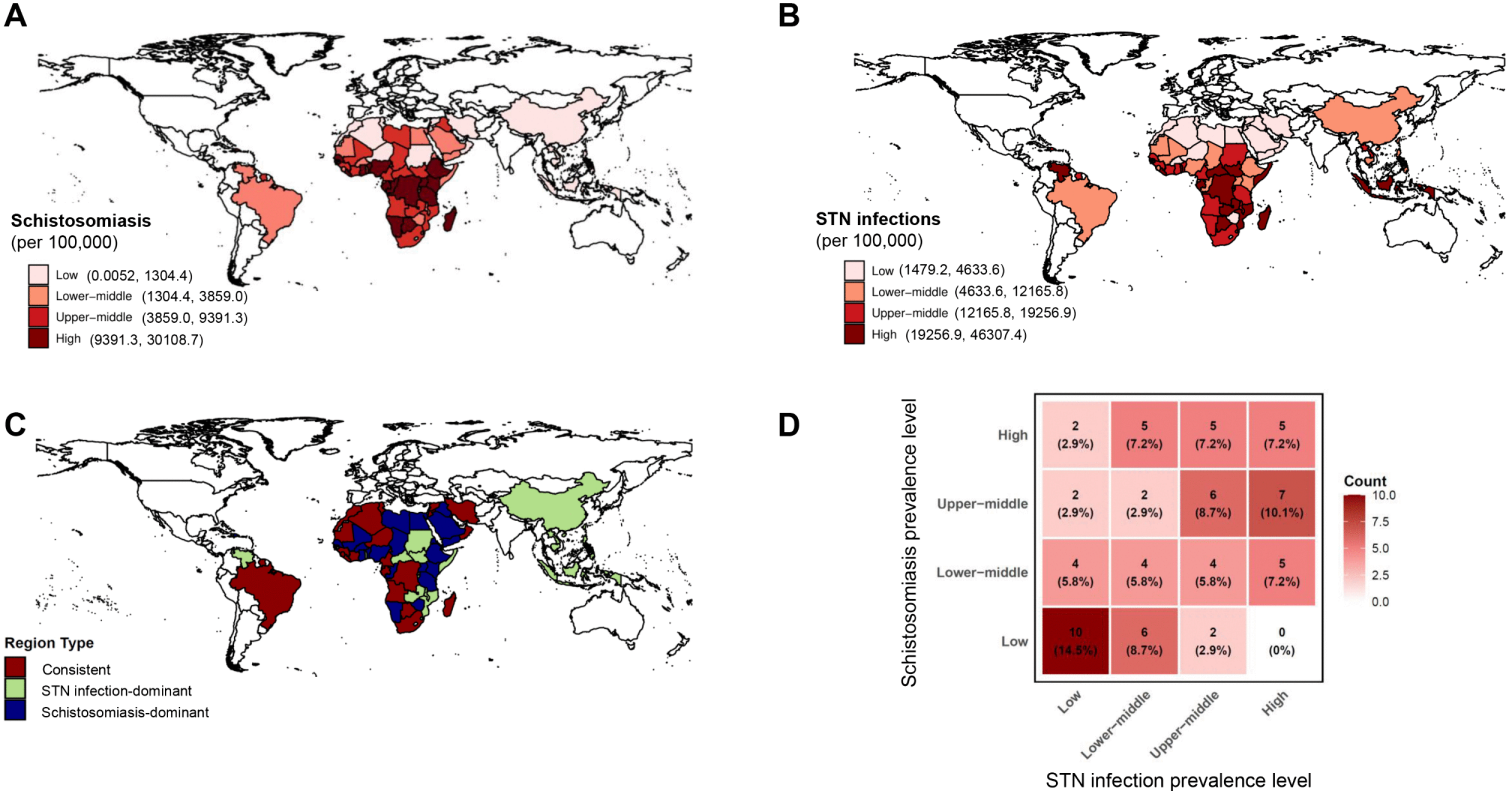
Global disease burden of schistosomiasis and STN infections in 2021. **(A)** Global distribution of schistosomiasis. **(B)** Global distribution of STN infections. **(C)** Global co-occurrence distribution of schistosomiasis and STN infections. **(D)** Heatmap depicting the co-occurrence distribution of countries and territories by disease prevalence levels of schistosomiasis and STN infections. Specific countries and territories in different co-occurrence pattern areas were listed in S3 Table. The basemap boundaries are derived from the Natural Earth project (https://www.naturalearthdata.com/) and were created using the R maps package.

To characterize the global co-burden of schistosomiasis and STN infections, we mapped their spatial distributions and co‑occurrence patterns using GBD‑derived prevalence data from 2021. Globally, upper-middle to high prevalence of schistosomiasis was concentrated in sub‑Saharan Africa, with scattered lower-middle prevalence in parts of Latin America, North Africa, and the Middle East (Fig 1A). As expected, STN infections showed a similar prevalence distribution. Sub‑Saharan Africa and Southeast Asia were the core high‑burden regions, while lower-middle prevalence was observed in Latin America and East Asia (Fig 1B).

Because the GBD database provides schistosomiasis prevalence data for only 69 countries and territories which were listed in S2 Table, our co‑occurrence analysis was restricted to these locations. Countries and territories were classified into three co‑occurrence patterns: consistent regions, schistosomiasis‑dominant regions, and STN infection‑dominant regions. Geographically, consistent regions were mainly clustered in sub‑Saharan Africa, the Middle East, and Latin America. Schistosomiasis‑dominant and STN infection‑dominant patterns were scattered across Africa, the Middle East, Southeast Asia, and East Asia (Fig 1C). We further quantified co‑occurrence by cross‑tabulating the prevalence levels of the two diseases. Among the 69 countries analyzed in this study, most countries (47, 68.1%) fell into the upper‑right and lower‑left quadrants, where both diseases showed either high (23, 33.3%) or low (24, 34.8%) prevalence. Fewer countries (22, 31.9%) exhibited discordant prevalence levels (e.g., no countries had high schistosomiasis but low STN infections) (Fig 1D).

### Long-term co-occurrence dynamics of the two parasitic diseases

**Fig 2:**
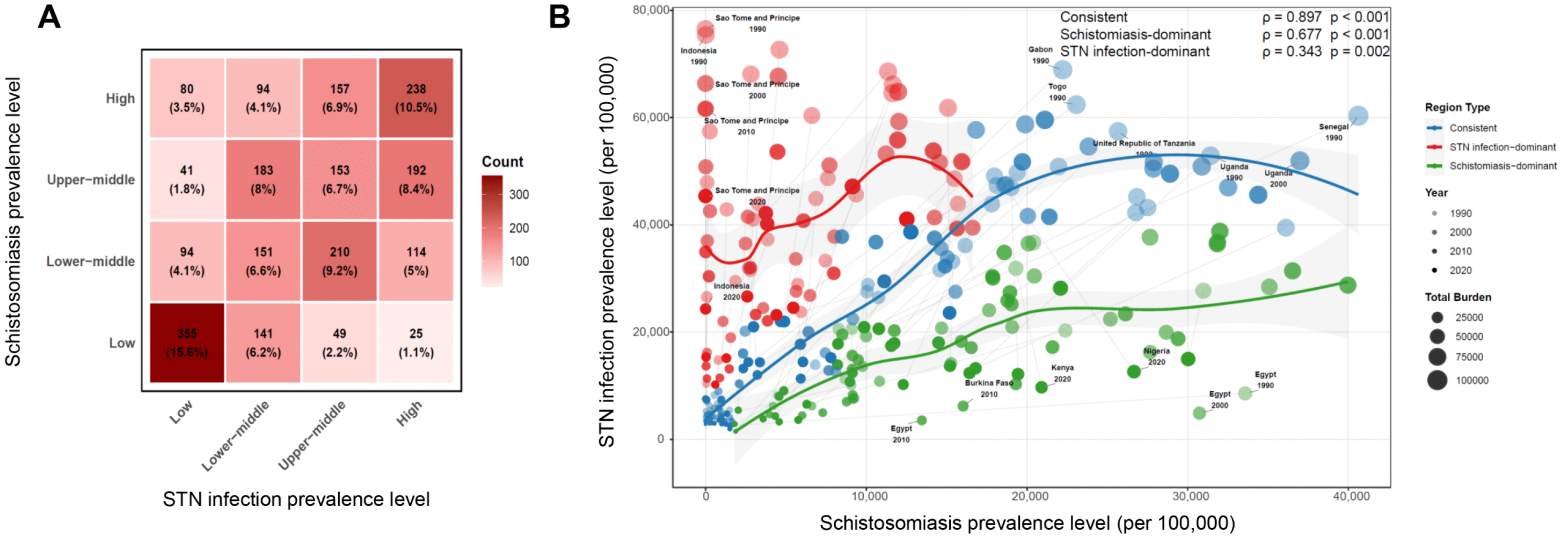
Spatio-temporal co-occurrence profiles of schistosomiasis and STN infections globally (1990–2021). **(A)** Heatmap depicting the co-occurrence distribution of schistosomiasis and STN infections. **(B)** Temporal evolution of schistosomiasis-STN infection comorbidity patterns.

We further characterized the global long-term co-burden of schistosomiasis and STN infections using prevalence data derived from the GBD Study spanning 1990–2021. Over this 32-year period, a total of 2208 comorbidity data for the two diseases were generated across 69 countries and territories. Among these 2208 data, the majority (1481, 67.0%) fell within the upper right and lower left quadrants of the disease prevalence heatmap, where both diseases exhibited either high (740, 33.5%) or low (741, 33.5%) prevalence (Fig 2A). These results were fully consistent with the data distribution observed in 2021 alone, indicating that the comorbidity relationship between the two diseases remained stable over the 32-year period.

We also analyzed the temporal trajectories of the global disease burden of these two parasitic diseases from 1990 to 2021, stratified by three predefined co-occurrence region types (Fig 2B). Regions dominated by STN infections experienced a marked increase in disease burden (from approximately 30,000 to over 60,000 per 100,000 population), accompanied by a sustained moderate burden of schistosomiasis. In contrast, regions dominated by schistosomiasis showed a relatively low but gradual increase in disease burden (from near 0–40,000 per 100,000 population), coupled with a stably low burden of STN infections. Consistent regions maintained coordinated moderate-to-high burdens for both diseases with minimal directional variation.

### Risk factor analysis for schistosomiasis and STN infections

We employed negative binomial regression to quantify the association between 22 selected risk factors (extracted from the GBD database) and the prevalence of schistosomiasis and STN infections. For schistosomiasis, few risk factors showed statistically significant associations (Table 1). No access to handwashing facility was associated with a small but significant increase in risk (RR 1.010, 95% CI 1.000–1.020, *P* = 0.039). High temperature (RR 0.980, 95% CI 0.968–0.993, *P* = 0.002) and low temperature (RR 0.970, 95% CI 0.954–0.987, *P* = 0.001) were inversely associated with schistosomiasis risk. Diet high in trans fatty acids also showed a marginal protective effect (RR 0.972, 95% CI 0.952–1.002, *P* = 0.045). For STN infections, multiple risk factors demonstrated significant associations (Table 1). Iron deficiency (RR 1.050, 95% CI 0.993–1.112, *P* = 0.038), zinc deficiency (RR 1.030, 95% CI 1.015–1.044, *P* < 0.001), diet low in vegetables (RR 1.025, 95% CI 1.016–1.034, *P* < 0.001), diet low in milk (RR 1.017, 95% CI 1.002–1.030, *P* = 0.040), diet low in omega-6 polyunsaturated fatty acids (RR 1.007, 95% CI 1.001–1.013, *P* = 0.036), diet low in calcium (RR 1.013, 95% CI 1.005–1.021, *P* = 0.001), no access to handwashing facility (RR 1.012, 95% CI 1.007–1.018, *P* < 0.001), child growth failure (RR 1.042, 95% CI 0.994-1.094, *P* = 0.043), unsafe water source (RR 1.017, 95% CI 1.009–1.024, *P* < 0.001), and unsafe sanitation (RR 1.013, 95% CI 1.007–1.019, *P* < 0.001) were all associated with increased risk. Protective factors included diet high in trans fatty acids (RR 0.977, 95% CI 0.962–0.998, *P* = 0.007), high temperature (RR 0.979, 95% CI 0.973–0.986, *P* < 0.001), low temperature (RR 0.977, 95% CI 0.969–0.987, *P* < 0.001), and low birth weight and short gestation (RR 0.969, 95% CI 0.946–0.995, *P* = 0.007).

**Table 1:** Relative risk (RR) and 95% confidence interval (CI) of risk factors affecting schistosomiasis and STN infections.

Risk Factor	Schistosomiasis		STN infections	
	RR (95% CI)	*P* value	RR (95% CI)	*P* value
Iron deficiency	1.042 (0.947, 1.156)	0.299	1.050 (0.993, 1.112)	0.038
Vitamin A deficiency	0.999 (0.970, 1.033)	0.967	1.017 (0.999, 1.036)	0.064
Zinc deficiency	1.013 (0.987, 1.040)	0.269	1.030 (1.015, 1.044)	0.000
Diet low in fruits	1.009 (0.990, 1.027)	0.371	1.007 (0.995, 1.019)	0.199
Diet low in vegetables	1.014 (0.995, 1.034)	0.074	1.025 (1.016, 1.034)	0.000
Diet low in whole grains	1.000 (0.985, 1.015)	0.955	1.001 (0.991, 1.012)	0.816
Diet low in nuts and seeds	1.002 (0.991, 1.013)	0.747	1.000 (0.993, 1.006)	0.919
Diet low in milk	1.011 (0.983, 1.034)	0.438	1.017 (1.002, 1.030)	0.040
Diet low in fiber	0.992 (0.977, 1.010)	0.323	1.005 (0.996, 1.015)	0.273
Diet low in seafood omega-3 fatty acids	0.998 (0.985, 1.011)	0.720	1.003 (0.995, 1.010)	0.511
Diet low in omega-6 polyunsaturated fatty acids	1.000 (0.989, 1.009)	0.972	1.007 (1.001, 1.013)	0.036
Diet high in trans fatty acids	0.972 (0.952, 1.002)	0.045	0.977 (0.962, 0.998)	0.007
Diet high in sodium	1.000 (0.972, 1.037)	0.990	1.006 (0.987, 1.028)	0.513
Diet low in calcium	1.004 (0.990, 1.019)	0.567	1.013 (1.005, 1.021)	0.001
No access to handwashing facility	1.010 (1.000, 1.020)	0.039	1.012 (1.007, 1.018)	0.000
Child growth failure	1.015 (0.934, 1.109)	0.670	1.042 (0.994, 1.094)	0.043
Diet low in legumes	1.000 (0.984, 1.017)	0.959	1.009 (0.999, 1.018)	0.072
High temperature	0.980 (0.968, 0.993)	0.002	0.979 (0.973, 0.986)	0.000
Low temperature	0.970 (0.954, 0.987)	0.001	0.977 (0.969, 0.987)	0.000
Low birth weight and short gestation	1.009 (0.966, 1.061)	0.662	0.969 (0.946, 0.995)	0.007
Unsafe water source	1.009 (0.996, 1.021)	0.189	1.017 (1.009, 1.024)	0.000
Unsafe sanitation	1.009 (0.998, 1.019)	0.098	1.013 (1.007, 1.019)	0.000

The PAFs were calculated for selected risk factors significantly associated with schistosomiasis and STN infections based on prior negative binomial regression analysis and supporting biomedical literature. For schistosomiasis, no access to handwashing facility, the only selected factor, contributed to 1.01% of cases. In regions where schistosomiasis is dominant, this factor accounted for 0.24% of cases, while in regions where both diseases are consistently present, its attributable fraction was 2.58% (Table 2). For STN infections, multiple factors showed notable PAFs (Table 2). In all regions combined, the leading contributors were iron deficiency (4.73%), child growth failure (4.06%), zinc deficiency (2.89%), diet low in vegetables (2.43%), and unsafe water source (1.64%). The combined burden of all evaluated risk factors was substantial. Regional patterns differed: in STN infection-dominant regions, iron deficiency remained the largest contributor (3.13%), whereas in consistent regions where both diseases coexist, iron deficiency accounted for 19.08% of cases, followed by child growth failure (8.82%) and zinc deficiency (4.57%).

**Table 2:** Population attributable fractions for schistosomiasis and STN infections of various risk factors.

Risk Factor	All regions		Schistosomiasis dominant regions		STN infections dominant regions		Consistent regions	
	Schistosomiasis	STN infections	Schistosomiasis	STN infections	Schistosomiasis	STN infections	Schistosomiasis	STN infections
Iron deficiency	NA	4.73	NA	0.00	NA	3.13	NA	19.08
Zinc deficiency	NA	2.89	NA	0.80	NA	1.67	NA	4.57
Child growth failure	NA	4.06	NA	0.00	NA	0.00	NA	8.82
Diet low in vegetables	NA	2.43	NA	0.00	NA	1.92	NA	3.60
Diet low in calcium	NA	1.32	NA	0.20	NA	0.06	NA	2.41
Diet low in milk	NA	1.64	NA	0.67	NA	0.50	NA	1.65
No access to handwashing facility	1.01	1.22	0.24	0.61	2.23	0.82	2.58	2.09
Unsafe water source	NA	1.64	NA	0.57	NA	1.06	NA	2.69
Unsafe sanitation	NA	1.32	NA	0.45	NA	0.70	NA	2.28
ALL	1.01	21.25	0.24	3.30	2.23	9.85	2.58	47.20

### Spatiotemporal variations in the evolution of disease burden across different regions

**Fig 3:**
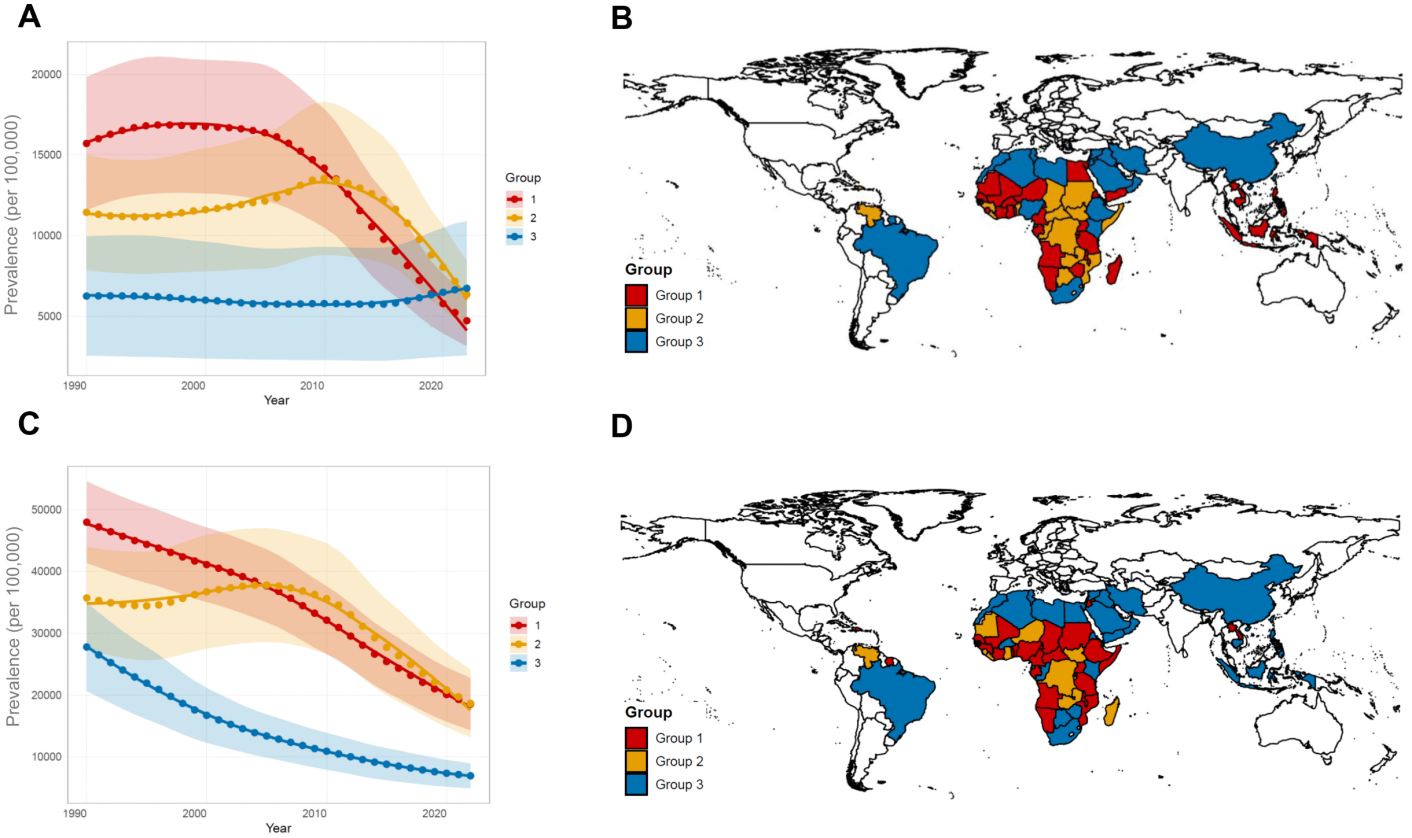
Group-based trajectory modelling (GBTM) of schistosomiasis and STN infection prevalence (1990–2021). **(A)** GBTM of schistosomiasis prevalence. Three distinct trajectory groups were identified: Group 1 (red): High and stable prevalence followed by rapid decline; Group 2 (green): Moderate prevalence with gradual increase then sharp decline; Group 3 (blue): Consistently low and stable prevalence. **(B)** Global distribution of three trajectories for schistosomiasis. Countries are color-coded according to their group assignment (Groups 1–3). **(C)** GBTM of STN infection prevalence. Three distinct trajectory groups were identified: Group 1 (red): Marked decline from high initial prevalence; Group 2 (green): Moderate stable prevalence followed by rapid decline; Group 3 (blue): Low and declining trend. **(D)** Global distribution of three trajectories for STN infection. Countries are color-coded according to their group assignment (Groups 1–3). The basemap boundaries are derived from the Natural Earth project (https://www.naturalearthdata.com/) and were created using the R maps package.

Using GBTM on GBD data from 1990 to 2021, we identified distinct evolutionary patterns in disease burden across regions. For schistosomiasis, three trajectories emerged (Fig 3A). Group 1 demonstrated a high prevalence that remained stable before declining rapidly; group 2 showed moderate prevalence that increased gradually before declining sharply; and group 3 exhibited consistently low and stable prevalence. Geographically, group 1 included sub-Saharan Africa and Southeast Asia; group 2 was concentrated selected nations in sub-Saharan Africa; and group 3 primarily comprised countries in East Asia, South America, North Africa, and the Middle East (Fig 3B). For STN infections, three distinct trajectories were also identified (Fig 3C). Group 1 showed a marked decline from a high initial prevalence; group 2 was characterized by moderate prevalence that remained stable before declining rapidly; and group 3 displayed a relatively low and declining trend. Geographically, Group 1 and 2 were concentrated in sub-Saharan Africa; Group 3 clustered in China, Southeast Asia, the Middle East, North Africa, parts of sub-Saharan Africa, and Brazil (Fig 3D).

### Global epidemiological clustering of schistosome-STN co-occurrence patterns

**Fig 4:**
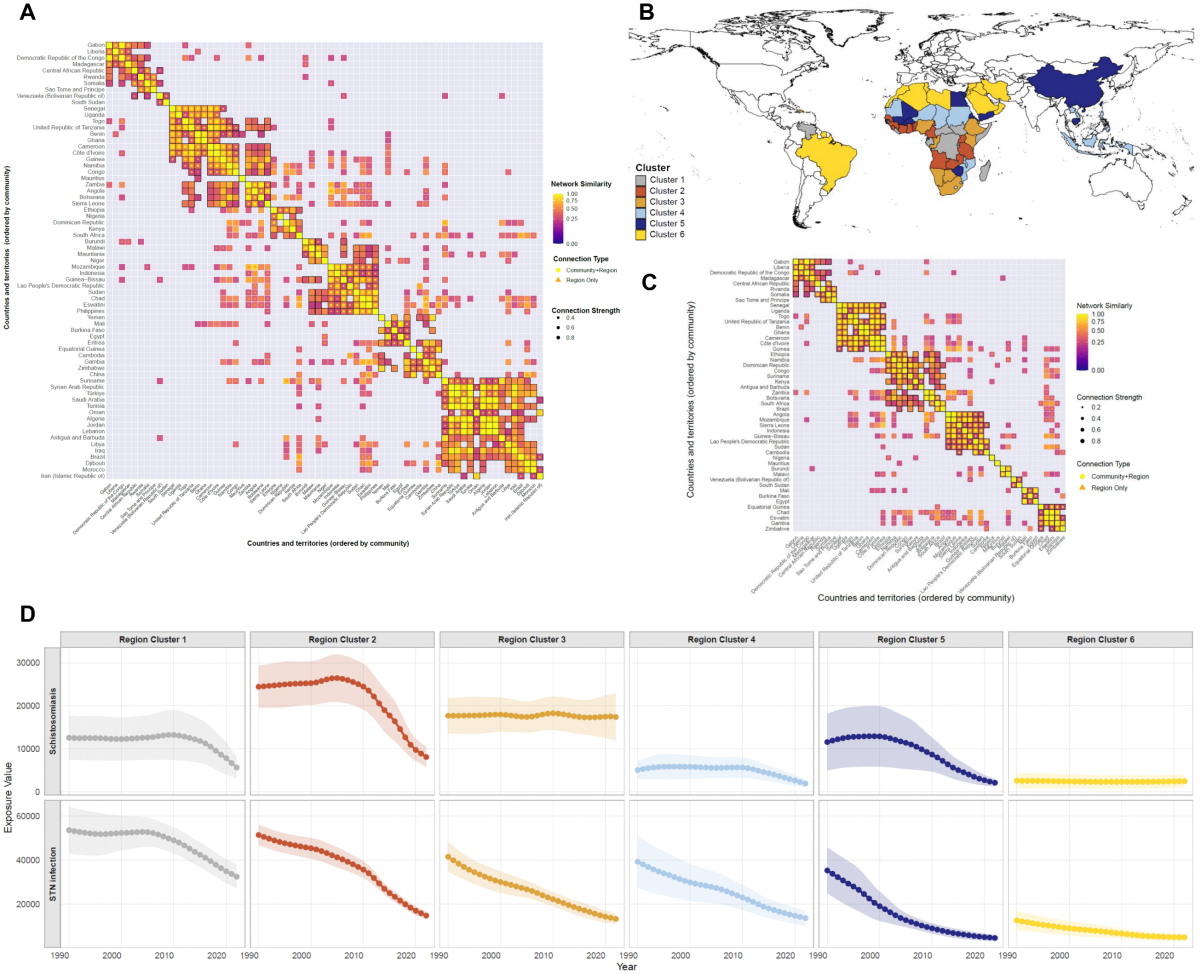
Country-level similarity network analysis for schistosomiasis-STN infection comorbidity patterns (1990–2021). **(A)** Clustered heatmap of pairwise country similarity. Rows and columns represent individual countries (n = 69). Color intensity (from blue to yellow) denotes the degree of similarity in comorbidity prevalence trajectories, with yellow indicating higher similarity. Countries are hierarchically clustered based on their 32-year co-occurrence patterns. **(B)** Global distribution of 6 distinct clusters. Countries are color-coded according to their cluster assignment (Clusters 1–6). **(C)** Zoomed-in view of the top 50 most similar country pairs from Panel **A.** This panel highlights the 50 country pairs with the highest similarity scores from Panel A, illustrating the strongest epidemiological connections across nations. **(D)** Comorbidity prevalence trajectories of schistosomiasis and STN infections across six global region clusters. For each cluster, the 32-year prevalence trajectories of schistosomiasis and STN infections are shown, with shaded areas representing 95% confidence intervals. This visualization confirms the distinct co-occurrence dynamics that define each cluster. The basemap boundaries are derived from the Natural Earth project (https://www.naturalearthdata.com/) and were created using the R maps package.

We conducted a country-level similarity network analysis of the 32-year (1990–2021) prevalence patterns of schistosomiasis and STN infections (Fig 4). These results partitioned the 69 countries and territories into 6 distinct epidemiological clusters: Cluster 1 (sub-Saharan African regions and Venezuela) exhibited coordinated, moderately high but declining burdens for both diseases; Cluster 2 (sub-Saharan African regions) showed steep, synchronized reductions in both infections; Cluster 3 (sub-Saharan African regions) maintained stable, moderate-to-high comorbidity burdens over the period; Cluster 4 (scattered sub-Saharan African and Southeast Asian regions) demonstrated low, slowly declining parallel burdens; Cluster 5 (China, Thailand, Egypt, etc.) displayed coordinated, steep declines from high initial burdens; and Cluster 6 (scattered North African, Latin American, and Middle Eastern regions) characterized by near-eliminated schistosomiasis and stable low STN infection.

## Discussion

This study systematically characterized the global co-occurrence patterns, long-term trajectories, and key risk factors of schistosomiasis and STN infections using GBD data spanning 32 years (1990–2021). The core findings reveal six distinct epidemiological clusters of co-occurrence, with heterogeneous burden dynamics across global regions; WASH factors as shared drivers; nutritional deficiencies as more impactful for STN infections, especially in consistent regions; and pronounced geographic aggregation of trajectory patterns. These results address the critical gap in global-scale understanding of polyparasitism dynamics, complementing insights from localized community studies and providing a data-driven foundation for targeted public health action.

The identification of six epidemiological clusters reflects the complex interplay between disease ecology, socioeconomic development, and intervention efforts. Clusters with persistently high co-occurrence burdens, predominantly in sub-Saharan Africa, align with previous observations of entrenched transmission in low social-demographic index regions, where inadequate WASH infrastructure and limited access to preventive chemotherapy sustain dual parasitic exposure [23,24]. In contrast, clusters characterized by synchronized declines in both diseases—exemplified by China—mirror the substantial progress of integrated control programs. China’s national schistosomiasis elimination initiative, for instance, reduced cases by 49% between 2016 and 2023 through a combination of mass drug administration, snail control, and improved sanitation [25]. The cluster with near-eliminated schistosomiasis but heterogeneous STN burdens may highlight the divergent transmission requirements of the two parasite groups. Schistosomes’ reliance on aquatic environments makes them more responsive to targeted water interventions, while STNs’ resilient egg survival in soil sustains transmission even in regions with improved water access.

Consistent with prior research, WASH-related factors (lack of handwashing facilities, unsafe sanitation) emerged as the most impactful shared drivers of co-occurrence, underscoring the WHO’s emphasis on integrating WASH with preventive chemotherapy for neglected tropical disease (NTD) control [4,26]. The PAF results further clarify the differential contributions of risk factors: nutritional deficiencies (iron deficiency, child growth failure) exerted greater influence on STN burden, reflecting the bidirectional relationship between intestinal parasitism and malnutrition. STN infections impair nutrient absorption, while poor nutrition reduces host resistance to infection [27,28]. Notably, our study reveals that the contribution of nutritional factors to STN infection risk is significantly amplified in schistosomiasis co-endemic regions. This finding aligns with a community survey in Brazil, which demonstrated that concurrent STN and schistosome infections markedly elevate anemia risk compared to single infections, reflecting the synergistic adverse effects of polyparasitism on host nutritional status [29]. Notably, our study identifies both high and low temperatures as shared protective factors for schistosomiasis and STN infections. This aligns with parasitic ecology: extreme temperatures impair the survival of schistosome cercariae in water and STN eggs or larvae in soil, disrupting transmission cycles.

The findings hold significant implications for global NTD control, particularly in the context of the WHO 2021–2030 NTD Roadmap [30]. For high-burden clusters—where nutritional deficiency emerges as a key risk factor for STN infections, especially in schistosomiasis co-endemic regions—prioritizing integrated interventions is critical. Combining WASH improvements, sustained preventive chemotherapy, and nutritional support addresses both shared and STN-specific drivers of co-occurrence. Single-disease strategies are unlikely to break transmission cycles given these intertwined risks. Regions with declining dual burdens should strengthen surveillance to detect rebounds, as illustrated by China’s sensitive snail monitoring. For clusters with heterogeneous STN burdens post-schistosomiasis control, targeted sanitation interventions plus nutritional interventions will yield disproportionate STN reduction benefits. Globally, these cluster-specific strategies align with the Roadmap’s “localized solutions” emphasis, ensuring resources target highest-priority regions and risk factors.

This study has several limitations that should be considered when interpreting the results. First, GBD schistosomiasis data are available for only 69 countries and territories—a limitation inherent to the focal endemicity of schistosomiasis. While this set captures the vast majority of the global schistosomiasis burden, our findings on co-occurrence patterns are generalizable only to schistosomiasis-endemic settings and should not be extrapolated to regions where schistosomiasis is absent. Second, GBD data rely on modeling to complement sparse surveillance data in low-resource settings, which may introduce uncertainty in burden estimates for some countries [31]. Third, prevalence estimates are modelled and may not capture hyper-local transmission heterogeneity. Fourth, risk factor data are ecological and do not establish causality; residual confounding may persist. Finally, co-occurrence was defined spatially and temporally rather than at the individual level. Future studies incorporating individual-level co-infection data and molecular or immunological markers would help refine burden estimates. Overall, this study provides a comprehensive global portrait of schistosome-STN co-occurrence, reinforcing the need for integrated, context-adapted approaches to accelerate progress toward NTD elimination goals.

## Supporting information

S1 Table Complete list of all 204 countries or territories included in this study. (DOCX)

S2 Table Complete list of all 69 countries or territories with schistosomiasis prevalence data. (DOCX)

S3 Table Countries and territories included in co-occurrence pattern analysis. (DOCX)
