# Supplementary material for: Epidemiology and risk factors of soil-transmitted nematode-schistosome co-occurrence: An analysis of the global burden of disease study
Source: PLoS Negl Trop Dis. 2026 May 4;20(5):e0014224. doi: 10.1371/journal.pntd.0014224 (PMC13138641; doi:10.1371/journal.pntd.0014224)
Supplement: S1 Table — (DOCX) [file pntd.0014224.s001.docx]

**S1 Table** . Complete list of all 204 countries or territories included in this study.

| **Country or territory** | **Number** |
| --- | --- |
| Myanmar, Malaysia, Maldives, Democratic People's Republic of Korea, Taiwan (Province of China), Kyrgyzstan, Fiji, Turkmenistan, Marshall Islands, Armenia, Azerbaijan, Papua New Guinea, Kazakhstan, Vanuatu, Mongolia, Kiribati, Tajikistan, Timor-Leste, Tonga, Viet Nam, Samoa, Solomon Islands, Sri Lanka, Georgia, Micronesia (Federated States of), Thailand, Republic of Korea, Austria, Andorra, Belgium, Australia, Singapore, Latvia, New Zealand, Serbia, Japan, North Macedonia, Bosnia and Herzegovina, Bulgaria, Poland, Ukraine, Estonia, Slovenia, Brunei Darussalam, Czechia, Russian Federation, Slovakia, Cyprus, Montenegro, Lithuania, Hungary, Romania, Republic of Moldova, Uzbekistan, Belarus, Croatia, Albania, Denmark, Finland, France, Bahamas, Dominica, Switzerland, Iceland, Luxembourg, United States of America, Malta, Argentina, Belize, Spain, Italy, Cuba, Germany, Ireland, Barbados, Greece, Portugal, Haiti, Sweden, Uruguay, Chile, Guyana, Netherlands, Canada, Grenada, United Kingdom, Jamaica, Norway, Israel, Saint Lucia, Guatemala, Paraguay, Panama, Honduras, Saint Vincent and the Grenadines, Peru, El Salvador, Bolivia (Plurinational State of), Ecuador, Nicaragua, Mexico, Colombia, Bahrain, Trinidad and Tobago, Costa Rica, Kuwait, Qatar, Palestine, Bangladesh, United Arab Emirates, Nepal, Afghanistan, Comoros, Pakistan, Bhutan, Seychelles, India, Lesotho, Cabo Verde, American Samoa, Cook Islands, Bermuda, Greenland, Guam, Northern Mariana Islands, Saint Kitts and Nevis, Puerto Rico, Monaco, Nauru, Tokelau, San Marino, Tuvalu, Niue, United States Virgin Islands, Palau, Sao Tome and Principe, Cambodia, Lebanon, Indonesia, Lao People's Democratic Republic, Djibouti, Iran (Islamic Republic of), Algeria, Jordan, Torkiye, Philippines, Morocco, Chinese Mainland, Tunisia, Syrian Arab Republic, Niger, Sudan, Oman, Burkina Faso, Egypt, Yemen, Saudi Arabia, Equatorial Guinea, Sierra Leone, Burundi, Eswatini, Rwanda, Venezuela (Bolivarian Republic of), Malawi, Brazil, Zimbabwe, Somalia, Togo, Eritrea, Mauritania, Mozambique, Guinea-Bissau, Gambia, Iraq, Chad, South Sudan, Mali, Angola, Libya, South Africa, Guinea, Cameroon, Cote d'Ivoire, Antigua and Barbuda, Central African Republic, Suriname, Zambia, Democratic Republic of the Congo, Senegal, Madagascar, United Republic of Tanzania, Namibia, Gabon, Congo, Benin, Botswana, Uganda, Dominican Republic, Ghana, Liberia, Ethiopia, Kenya, Nigeria, Mauritius | 204 |
