# Supplementary material for: Epidemiology and risk factors of soil-transmitted nematode-schistosome co-occurrence: An analysis of the global burden of disease study
Source: PLoS Negl Trop Dis. 2026 May 4;20(5):e0014224. doi: 10.1371/journal.pntd.0014224 (PMC13138641; doi:10.1371/journal.pntd.0014224)
Supplement: S2 Table — (DOCX) [file pntd.0014224.s002.docx]

**S2 Table**. Complete list of all 69 countries or territories with schistosomiasis prevalence data.

| **Country or territory** | **Number** |
| --- | --- |
| Sao Tome and Principe, Cambodia, Lebanon, Indonesia, Lao People's Democratic Republic, Djibouti, Iran (Islamic Republic of), Algeria, Jordan, Torkiye, Philippines, Morocco, Chinese Mainland, Tunisia, Syrian Arab Republic, Niger, Sudan, Oman, Burkina Faso, Egypt, Yemen, Saudi Arabia, Equatorial Guinea, Sierra Leone, Burundi, Eswatini, Rwanda, Venezuela (Bolivarian Republic of), Malawi, Brazil, Zimbabwe, Somalia, Togo, Eritrea, Mauritania, Mozambique, Guinea-Bissau, Gambia, Iraq, Chad, South Sudan, Mali, Angola, Libya, South Africa, Guinea, Cameroon, Cote d'Ivoire, Antigua and Barbuda, Central African Republic, Suriname, Zambia, Democratic Republic of the Congo, Senegal, Madagascar, United Republic of Tanzania, Namibia, Gabon, Congo, Benin, Botswana, Uganda, Dominican Republic, Ghana, Liberia, Ethiopia, Kenya, Nigeria, Mauritius | 204 |
