# Supplementary material for: Epidemiology and risk factors of soil-transmitted nematode-schistosome co-occurrence: An analysis of the global burden of disease study
Source: PLoS Negl Trop Dis. 2026 May 4;20(5):e0014224. doi: 10.1371/journal.pntd.0014224 (PMC13138641; doi:10.1371/journal.pntd.0014224)
Supplement: S3 Table — (DOCX) [file pntd.0014224.s003.docx]

**S3 Table**. Countries and territories included in co-occurrence pattern analysis.

| **Co-occurrence pattern** | **Country or territory** | **Number** |
| --- | --- | --- |
| Consistent | Algeria, Brazil, Iran (Islamic Republic of), Jordan, Suriname, Lebanon, Oman, Morocco, Syrian Arab Republic, Madagascar, Türkiye, Gabon, Tunisia, Angola, Equatorial Guinea, Democratic Republic of the Congo, South Africa, Guinea, Cameroon, Côte d'Ivoire, Botswana, Sierra Leone, Niger, Liberia, Mauritania | 25 |
| Schistosomiasis dominant | Antigua and Barbuda, Dominican Republic, Egypt, Iraq, Libya, Saudi Arabia, Mauritius, Ethiopia, Congo, Yemen, Eritrea, Kenya, Uganda, United Republic of Tanzania, Chad, Namibia, Benin, Burkina Faso, Ghana, Gambia, Zimbabwe, Nigeria, Senegal, Mali | 24 |
| STN infection dominant | Philippines, Cambodia, Chinese Mainland, Indonesia, Lao People's Democratic Republic, Venezuela (Bolivarian Republic of), Burundi, Central African Republic, Djibouti, Malawi, Somalia, Rwanda, Zambia, Mozambique, Eswatini, Guinea-Bissau, Sao Tome and Principe, Togo, South Sudan, Sudan | 20 |
